# Homing in hematopoietic stem cells: focus on regulatory role of CXCR7 on SDF1a/CXCR4 axis

**DOI:** 10.17179/excli2014-585

**Published:** 2016-02-15

**Authors:** Amir Asri, Javid Sabour, Amir Atashi, Masoud Soleimani

**Affiliations:** 1 Department of Hematology, Faculty of Medical Sciences, Tarbiat Modares University, Tehran, Iran

**Keywords:** CXCR7, CXCR4, CXCL12, HSCs homing

## Abstract

Hematopoietic stem cells (HSCs) form a rare population of multipotent stem cells, which give rise to all hematopoietic lineages. HSCs home to bone marrow niches and circulate between blood and bone marrow. Many factors, especially SDF1a, affect the circulation of HSCs, but these have not been fully recognized. SDF1a has been shown to bind CXCR7 in addition to CXCR4 and can also function as SDF1a/CXCR4 modulator. CXCR7 plays a role in HSCs homing via SDF1a gradient and is a mediator of CXCR4/SDF1a axis. This review describes the current concepts and questions concerning CXCR7/CXCR4/SDF1a axis as an important key in hematopoietic stem cells homing with particular emphasis on CXCR7 receptor. Homing of HSCs is an essential step for successful hematopoietic stem cell transplantation.

## Introduction

Hematopoietic stem cells migrate through blood circulation and bone marrow niches. HSCs migration to bone marrow is an essential step in clinical stem cell engraftment. HSCs are a rare population of multipotent stem cells lodging in a specialized microenvironment within the bone marrow known as bone marrow niche. Homing of HSCs starts from the embryonic period, when HSCs migrate from the fetal liver to seed bone marrow via crosstalk between cytokines and adhesion molecules, especially SDF1a, CXCR4 and CXCR7 pathways (McGrath et al., 1999[33]; Gazitt, 2004[14]). SDF1a chemokine and its receptors have been implicated in survival, proliferation and trafficking of CD34+ cells (Lataillade et al., 2000[26]; Aiuti et al., 1997[1]; Mohle et al., 1998[35]; Wright et al., 2002[66]). CXCR4 was deemed to be the sole receptor for SDF1a but CXCR7 was later found to be another high affinity receptor for SDF1a (Balabanian et al., 2005[2]; Burns et al., 2006[6]). It is unclear whether the SDF1a/CXCR7 axis regulates HSCs homing, but there are evidences that CXCR7 modulates CXCR4/SDF1 responses (Hartmann et al., 2008[18]; Naumann et al., 2010[38]; Uto-Konomi et al., 2013[59]).

## Bone Marrow Niches and Homing of HSCs

HSCs are multipotent stem cells capable of generating all hematopoietic lineages. The bone marrow cavities are the main residence for post-natal human hematopoiesis. HSCs must be maintained in a specific niche, which is critical for HSCs fate and hematopoiesis. HSCs niche, which is an inductive microenvironment, provides factors that maintain the hematopoietic stem cells and prevent their differentiation (Schofield, 1978[48]). Bone marrow niche maintains several functions of HSCs, including stem cell adhesion, self-renewal (Jones and Wagers, 2008[22]), apoptosis (Lin, 2002[29]) as well as homing and mobilization. HSCs physiologically leave the bone marrow and migrate between blood circulation and bone marrow niche (Wright et al., 2001[67]), a process termed mobilization and homing. Many agents have been reported to affect the homing of HSCs, most notably integrins (Watanabe et al., 1998[62]; Qian et al., 2006[43]), selectins (Sullivan et al., 2011[55]) and chemokines (Aiuti et al., 1997[1]). CXCR7/ CXCR4/SDF1a axis plays major roles in stem cell motility, which will be discussed later.

### SDF1 alpha/CXCR4 axis

SDF1a is expressed in bone tissue and endothelial cells and can induce the expression of LFA1 and VLA4 adhesion molecules. However, the expression of CXCR4 in USSCs is decreased with the increase in passage number. There are two isoforms of SDF1a, and its gene is located on human chromosome 10 (Levesque et al., 2003[27]; Hartmann et al., 2008[18]; Watt and Forde, 2008[63]). In most reports, CXCL12-abundant reticular cells (CARs) and osteoblasts are introduced as the main sources of SDF1a in both osteoblastic and vascular niches (Jung et al., 2006[23]; Sugiyama et al., 2006[54]). Cytokines and hormones play a role in SDF1a expression. For instance, TNF-α, IL-1 beta, PTH and PDGF-BB stimulate SDF1a secretion in vitro. However, TGF-beta 1 rapidly reduces SDF1a expression in most osteoblastic cell lines (Jung et al., 2006[23]). CXCR-4 (also identified as fusin or CD184) is the receptor for SDF1a chemokine. SDF1a/ CXCR4 axis plays a role in HSCs homing and quiescence (Moriuchi et al., 1997[37]; Saini et al., 2010[46]). Ex vivo pretreatment of HSCs with high dose SDF1a reduces CD34+ cells engraftment, but low dose SDF1a increases their engraftment. These diverse effects are likely to be mediated by various second messenger systems sensitive to different concentrations of SDF1a (Plett et al., 2002[42]). CXCR4 has a tendency to form hetero- and homo oligomers, and such oligomerization could play a role in the allosteric regulation of CXCR4 signaling (Wu et al., 2010[68]). CXCR4 signaling is mediated through activation of G proteins, AKT, ERK and JAK-STAT signaling pathway (Hartmann et al., 2008[18]; Teicher and Fricker, 2010[57]). Obstruction of CXCR4-SDF1a axis pathway leads to proliferation of HSCs (Naumann et al., 2010[38]). In contrast, some experiments have reported that SDF1a enhances the repopulating potential of CD34+cells, and have suggested that the function of hematopoietic progenitor cells (HPCs) can be satisfactorily regulated through specific CXCR4 signaling (Plett et al., 2002[42]). In BM cells, CXCR4 regulates the homing and presentation of circulating functional SDF1a; therefore, these cells regulate the trafficking of HSCs together with CXCR4 positive HSCs. Immature HSCs express low levels of SDF1a that can interact with CXCR4 on stromal cells by secretion of this ligand, reversing the usual form of interaction and activation (Dar et al., 2006[10]). Stem cell factor (SCF) and IL-6 enhance CXCR4 on the CD34+ cells and improve stem cell engraftment (Peled et al., 1999[41]). CXCR4 knockout mice have defective B lymphopoiesis and myelopoiesis and die perinatally, showing intense defects in the hematopoietic tissue (Ma et al., 1998[32]). HIF-1 plays a role in SDF1a and CXCR4 expression, and improves the function of CXCR4. Drugs such as Plerixafor (AMD3100) that block CXCR4 or G-CSF which causes elastase release from neutrophils and SDF1a degradation lead to HSCs mobilization (Balabanian et al., 2008[3]; Rajagopal et al., 2010[44])

### CXCR7 as a novel receptor for SDF1a

**Figure 1:**
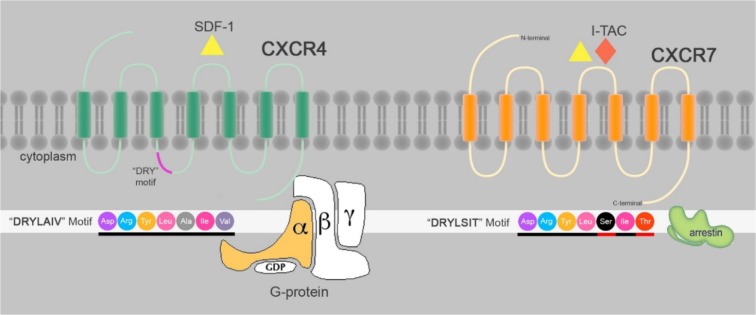
Schematic structure of CXCR4 and CXCR7: CXCR4 and CXCR7 are 7TMRs that share binding to SDF1a. CXCR7 also binds to I-TAC. “DRYLAIV” is a common motif in GPCRs modified to “DRYLSIT” motif essential for G-protein responses. SDF1a/CXCR7 interactions recruit Beta-arrestin but not G-protein.

Although CXCR4/SDF1a was thought to be a single axis, the interaction of SDF1a with CXCR4 was questioned after Burns report (Burns et al., 2006[6]). He showed that small molecular inhibitors of CCX451, CCX751 and CXCL11 compete with SDF1a binding to CXCR4, with human cancer cell lines showing a contradictory pattern of CXCR4 expression and SDF1a binding. Due this observation, he suggested a new receptor for SDF1a, the orphan receptor RDC-1, which was named CXCR7 (Burns et al., 2006[6]). CXCR7 (RDC-1; CXC-CK) is a seven-transmembrane-spanning chemokine protein receptor (Heesen et al., 1998[19]) with characteristic structural features similar to other chemokine receptors (Figure 1(Fig. 1)). However, it is a decoy CXC chemokine receptor similar to Duffy Antigen of D6 (Graham, 2009[16]). Decoy chemokine family is characterized by modification of Asp-Arg-Tyr-Leu-Ala-Ile-Val (DRYLAIV) motif essential for G-protein mediated responses (Haraldsen and Rot, 2006[17]; Graham, 2009[16]). CXCR7 does not signal through G proteins, and is an atypical chemokine receptor (Rajagopal et al., 2010[44]). Previously, CXCR7 was wrongly thought to be a receptor for vasoactive intestinal peptide (VIP) (Heesen et al., 1998[19]). CXCR7 is involved in several biological processes, including embryonic development (Burns et al., 2006[6]), scavenging for SDF1a and CXCL11 (Naumann et al., 2010[38]), promotion of adhesion to activated endothelial cells, cell survival but not cell proliferation or tumor development (Naumann et al., 2010[38]; Tarnowski et al., 2010[56]; Kumar et al., 2012[25]). Like CXCR4, CXCR7 is highly conserved in mice and humans (Heesen et al., 1998[19]), so that CXCR7 -/- mice show different heart abnormalities and die at birth (Sierro et al., 2007[52]). CXCR7 binds to SDF1a and I-TAC (IFN-inducible T cell chemo attractant; CXCR11) (Balabanian et al., 2005[2]; Burns et al., 2006[6]). Like CXCR4, CXCR7 N-terminal domain binds to SDF1a (Veldkamp et al., 2008[61]). CXCR7 is expressed in activated endothelial cells, tumor cell lines, early fetal liver cells, transforming cells (Burns et al., 2006[6]), migrating primordium in zebrafish (Dambly-Chaudiere et al., 2007[9]), monocytes, human fetal heart, neuronal tissue, adult mouse cerebellum, mature B cells (Infantino et al., 2006[21]; Thelen and Thelen, 2008[58]), blood and lymphatic endothelial cells in human renal allografts (Neusser et al., 2010[39]). Nevertheless, the expression of CXCR7 on several cell types is controversial (Balabanian et al., 2005[2]; Burns et al., 2006[6]; Kumar et al., 2012[25]), and it is poorly expressed on human and mouse T-cells and CD34+ progenitors (Infantino et al., 2006[21]; Hartmann et al., 2008[18]; Tarnowski et al., 2010[56]) but the expression of its mRNA has been shown in PBL (Shimizu et al., 2000[51]). CXCR7 is required for activation of LFA-1 and VLA-4 on T-cells and CD 34+ cells under shear flow (Hartmann et al., 2008[18]) but the role of CXCR7 in chemotaxis and cell migration has been a subject of debate. Some reports have suggested that CXCR7 induces T-cell migration (Balabanian et al., 2005[2]; Kumar et al., 2012[25]). In contrast, other experiments showed that CXCR7 is not involved in SDF1a/ CXCR4/CXCR7 mediated T-cell and CD34+ mobilization (Burns et al., 2006[6]; Hartmann et al., 2008[18]; Naumann et al., 2010[38]; Tarnowski et al., 2010[56]). Like CXCR4, CXCR7 is a co-receptor for several HIV and SIV strains (Shimizu et al., 2000[51]).The human CXCR7 gene was mapped to chromosome 2, closely linked to CXCR1, CXCR2, and CXCR4 genes (Balabanian et al., 2005[2]; Thelen and Thelen, 2008[58]). CXCR7 circulates between the cell and endosomal membrane in presence or absence of ligand, slightly reduced only after long term incubation with SDF1a and CXCL11, leading to ligand vanishing due to internalization. In contrast, after encounter with ligand, CXCR4 undergoes down regulation and ligand disappears to some extent. DYR sequence deletion does not affect the clearance of SDF1a but C-terminal deletion inhibits SDF1a internalization and wiping out (Naumann et al., 2010[38]).

### CXCR7 Recruitment of beta-arrestin but not G-protein

**Figure 2:**
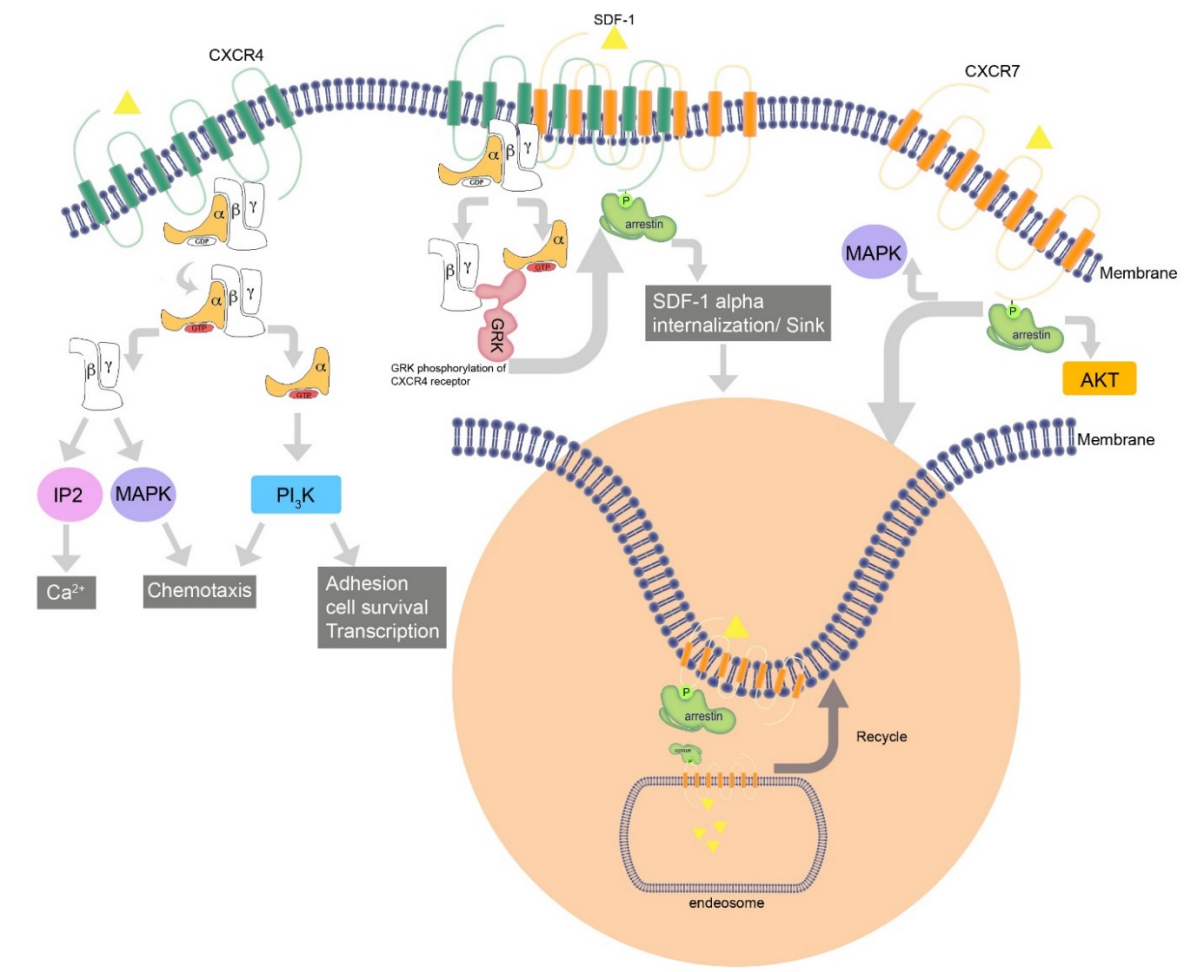
CXCR7, CXCR4 and CXCR7/CXCR4 hetrodimerization signaling pathways

Seven-transmembrane receptors (7TMRs) signal through G proteins and β-arrestins (Figure 2(Fig. 2)). The latter function as multipurpose adapters controlling receptor signaling, desensitization and trafficking. Commonly, there is a balance between G protein and beta-arrestin-mediated pathways. Researches revealed that CXCR7 does not couple to G proteins and cannot lead to GTP hydrolysis or calcium mobilization; nevertheless, it does trigger MAP kinases cascade through beta-arrestins (Rajagopal et al., 2010[44]). CXCR7 inhibition does not affect the degree or kinetics of CXCL12-triggered Akt or ERK1/2 phosphorylation. However, one study showed that CXCR7-SDF1a interaction activetes ERK protein and AKT in T-cells, which is Giα independent (Kumar et al., 2012[25]). In contrast, ERK and Akt activation is completely dependent upon CXCR4-triggered Gi signaling due to complete inhibition of it by AMD3100 and Gi- inhibitor PTX. Similar to other chemokines, ligand binding to CXCR7 regulates downstream signaling pathways. Unlike other chemokines, decoy receptor CXCR7 fails to activate G-proteins responses but it activates beta-arrestin pathway. Upon binding CXCR7, CXCL12 activates beta-arrestin2 and mediates pERK formation, which is activated by G-protein. β-arrestin depletion reduces the migration of rVSMC in response to I-TAC (Rajagopal et al., 2010[44]).

On the other hand, a research showed that co-expression of CXCR4 and CXCR7 in the same cell leads to strong SDF1a-induced intracellular calcium signaling. If CXCR7 sequesters arrestin2 away from CXCR4, this may eliminate signal-regulating attributes of the beta-arrestins, and allow for improved signaling through CXCR4 (Zabel et al., 2009[69]). It has been revealed that CXCR7 cooperates with beta-arrestin in a ligand and dose independent mode, which leads to ligand internalization. CXCR7 recruitment of beta-arrestin is associated with ERK phosphorylation in a short period of time, which is then distributed in cytoplasmic vesicles in comparison with G protein-mediated activation of pERK, which takes longer time and is dispensed to cytoplasm and nucleus (Rajagopal et al., 2010[44]). Recruitment of beta-arrestin is dependent upon C-terminal end of the receptor. SDF1a initiates beta-arrestin2 association with both CXCR4 and CXCR7. 

Beta-arrestin2 may create a distinct intracellular signaling pathway that can participate in cross-talk between CXCR7 and CXCR4. 

Heterodimerization of CXCR7 with CXCR4 changes the comparatively different orientation between CXCR4 and Giα, decreasing SDF1a mediated G protein activation.CXCR7 has an interaction with inactive G protein Gai1; nevertheless, cross-regulation of CXCR4 via G protein sequestration by CXCR7 has not been confirmed (Zabel et al., 2009[69]). 7TMRs include two groups: A and B receptors based on communication with beta-arrestin molecules (Moore et al., 2007[36]). Class A7TMRs have a temporary interaction with beta-arrestin and recycle quickly to the cell membrane from early endosomes, binding beta-arrestin2 versus beta-arrestin1 with high affinity. Class B 7TMRs show inverse properties and bind to both beta-arrestin 1 and 2. CXCR7 seems to have characteristics of both class A and class B 7TMRs (Luker et al., 2008[30]). It has considerably higher associations with beta-arrestin 2 similar to class A receptors, and there are complexes of CXCR7 and beta-arrestin2 that continue to increase over time as would be expected for a class B receptor (Chakera et al., 2008[7]). Noticeably, in the absence of SDF1a and under baseline situations, the interaction between CXCR7 and beta-arrestin2 is higher compared to control pairs of CXCR7 and c-fos or FKBP12 (Cheng et al., 2000[8]; Chakera et al., 2008[7]; Luker et al., 2009[31]). Luker et al. (2009[31]) reported that depletion of beta-arrestin in mouse embryonic fibroblasts reduces but does not eliminate the accumulation of SDF1a, suggesting that CXCR7 possibly uses another distinct pathway for uptake of SDF1a. Agents directed against CXCR4 such as AMD3100, which are used in saturating concentrations, could stimulate the association between CXCR7 and beta-arrestin2 in cells expressing both receptors.

### CXCR7 Interference with SDF1a/CXCR4 Axis responses

CXCR7 expression in B-cells is inversely correlated with the activation of CXCR4 (Levoye et al., 2009[28]). CXCR7 cannot intrinsically cause the signal to be transmitted. CXCR7 receptors in breast tumor cell line MCF-7 do not cause increased cytoplasmic Ca2+ or cell migration in response to SDF1a (Burns et al., 2006[6]), but Ca2+ mobilization and chemotaxis is triggered in transformed T-cell line expressing CXCL4/SDF1a. Although CXCR7 is not directly involved in migration of HSCP (hematopoietic stem cell progenitors) (Tarnowski et al., 2010[56]), its modulatory role in CXCR4/SDF1 axis has been mentioned in literature. 

CXCR7 can also act as a sink for SDF1a and regulates chemokine gradients (Naumann et al., 2010[38]; Hoffmann et al., 2012[20]). Hartmann et al. (2008[18]) suggested two pools of CXCR7 in CD34+ cells: the first expressed on cell surface and the second intracellular, the localization of which is associated with early endosomes. CXCR7 induces SDF1a internalization and degradation (Naumann et al., 2010[38]). CXCR4 heterodimerization with CXCR7 selectively impairs the ability of CXCR4 to activate G proteins after ligand binding, and alters CXCL12-CXCR7 axis mediated calcium responses and chemotaxis of T-cells (Levoye et al., 2009[28]).

### CXCR7 response is up-regulated by VLA-4 and LFA-1

The retention of adult HSCs in the bone marrow niche is controlled by several factors such as adhesion receptors, including integrins. Very late activation antigen-4 (VLA-4; α4β1) and lymphocyte function-associated antigen-1 (LFA-1; αLβ2) are integrin molecules involved in hematopoietic cell trafficking (Milner and Campbell, 2002[34]). LFA-1 and VLA-4 are expressed in HSCs (Williams et al., 1991[64]). Activation of LFA-1 and VLA-4 on lymphocytes is inhibited by PTX. SDF1a- triggers the activation of integrin through G protein-coupled chemokine receptor CXCR4, which is inactivated by PTX (Hartmann et al., 2008[18]). CXCR7 effects SDF1a-CXCR4-triggered activation of LFA-1 and VLA-4 in T-cells as well as CXCR4 and CXCR7 uptake, which is necessary for LFA-1 and VLA-4 activation in T-cells on TNF-α activated HUVEC (Hartmann et al., 2008[18]).The majority of CXCL12-triggered lymphocytes in inflammatory endothelial tissue are inhibited by CXCR7 blocking (Hartmann et al., 2008[18]). Moreover, antagonism of CXCR7 attenuates chronic hypoxia-induced pulmonary vascular adhesion (Sartina et al., 2012[47]). According to these investigations, high expression of integrin is shown to be efficient for homing and retention of lymphocytes (Gorfu et al., 2009[15]), macrophages (Patel et al., 1998[40]) and HSCs (Watanabe et al., 1998[62]; Fibbe et al., 2000[12]; Kronenwett et al., 2000[24]; Scott et al., 2003[50]; Rettig et al., 2012[45]). CXCR7 is required for integrin activation through CXCR4/SDF1a pathway, and CXCR7/SDF1a is a likely modulatory axis regulating CXCR4/SDF1a-mediated HSCs homing. Transfected CXCR7 positive cells are adhered to the activated HUVECs considerably higher than intact cells (Burns et al., 2006[6]). TNFα and IL-1β promote de novo CXCR7 expression on endothelial cells (Burns et al., 2006[6]).

## Conclusion

SDF1a/CXCR4 axis is regulated by many factors (Forde et al., 2007[13]; Williams et al., 2008[65]). According to previous content, CXCR7 is an important modulation factor. CXCR7 binds I-TAC and SDF1a, both of which can compete for binding to it. However, CXCR4 is a specific receptor for SDF1a (Burns et al., 2006[6]). I-TAC/CXCR7-medaited responses may interfere and compete with CXCR7-SDF1a mediated interaction (Burns et al., 2006[6]). CXCR4 expression is observed on HSCs and other cells. In contrast to CXCR4, CXCR7 is expressed on embryonic HSCs and cancer transformed cells. There is evidence that primitive erythrocytes express CXCR7 (Berahovich et al., 2010[4]) and traffic in embryonic CXCR7+ liver cells at E11-E13 in response to SDF1a (Burns et al., 2006[6]), so CXCR7 is likely to play a role in processes regulated by SDF1a. CXCR7 expression is up-regulated in some hematopoietic neoplasms like human B lymphoma (Burns et al., 2006[6]). CXCR7 blocking by antibody reduces the arrest of HSCs on immobilized VCAM-1 (Hartmann et al., 2008[18]). Zebra fish lateral-line primordium migration can be mediated only via CXCR7 (Valentin et al., 2007[60]). In addition, CXCR4 and CXCR7 are able to form heterodimer and homodimers and regulate CXCL12-promoted homing of HSCs (Levoye et al., 2009[28]). Some observations have demonstrated miRNA-mediated post translational regulation with CXCR7 and SDF1a expression (Staton et al., 2011[53]). Mutation in Von Hippel-Lindau (VHL), which degrades HIF1, leads to overexpression of CXCR4 (Burger and Kipps, 2006[5]). CXCR7 promoter contains two hypoxia-responsive elements (Tarnowski et al., 2010[56]). CXCR7 expression is up-regulated by hypoxia in human microvascular endothelial cells (Schutyser et al., 2007[49]; Esencay et al., 2013[11]). Overall, CXCR7/ SDF1a axis suggested a new migration path in homing of HSCs, and can be useful for therapeutic purposes.
